# Supplementary material for: Leveraging synthetic data produced from museum specimens to train adaptable species classification models
Source: PLoS One. 2025 Sep 3;20(9):e0329482. doi: 10.1371/journal.pone.0329482 (PMC12407421; doi:10.1371/journal.pone.0329482)
Supplement: S1 File — (PDF) [file pone.0329482.s001.pdf]

Supplemental

Figs

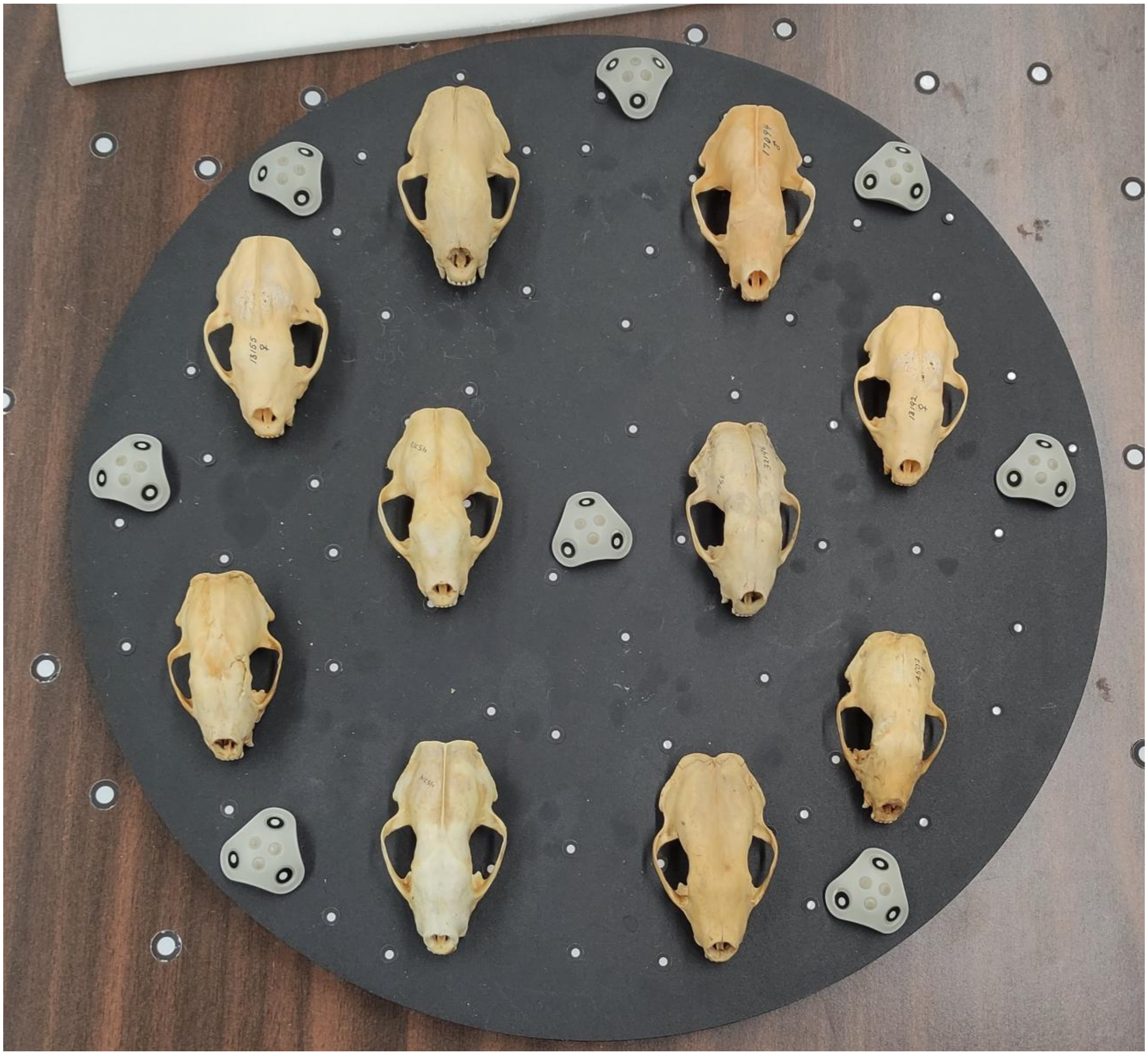

Fig S.1: A photograph of the skull 3D scanning table with 10 skunk (*Mephitis mephitis*) skulls.

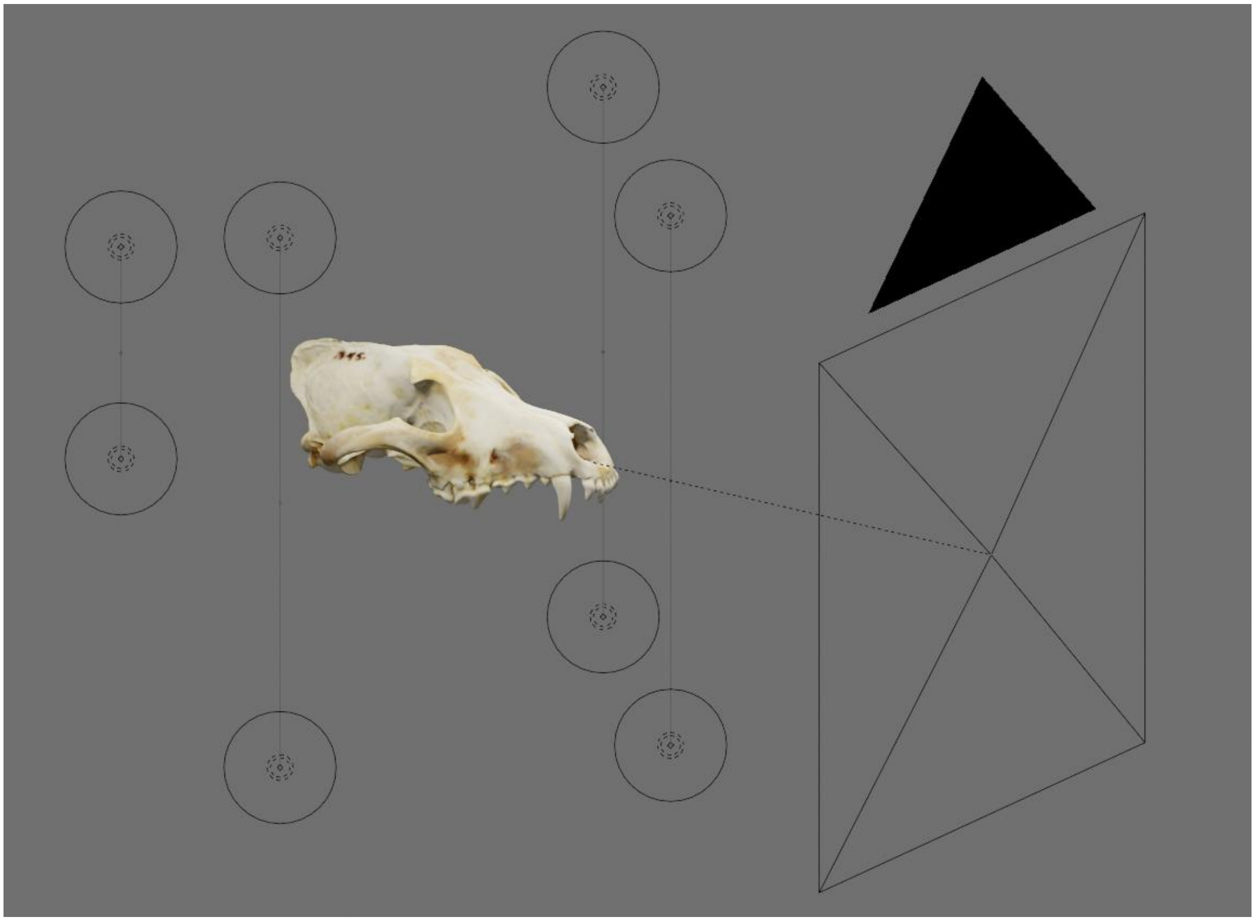

Fig S.2: The Blender environment with a wolf (*Canis lupus*) skull loaded. The background was brightened to enhance contrast of the user interface elements.

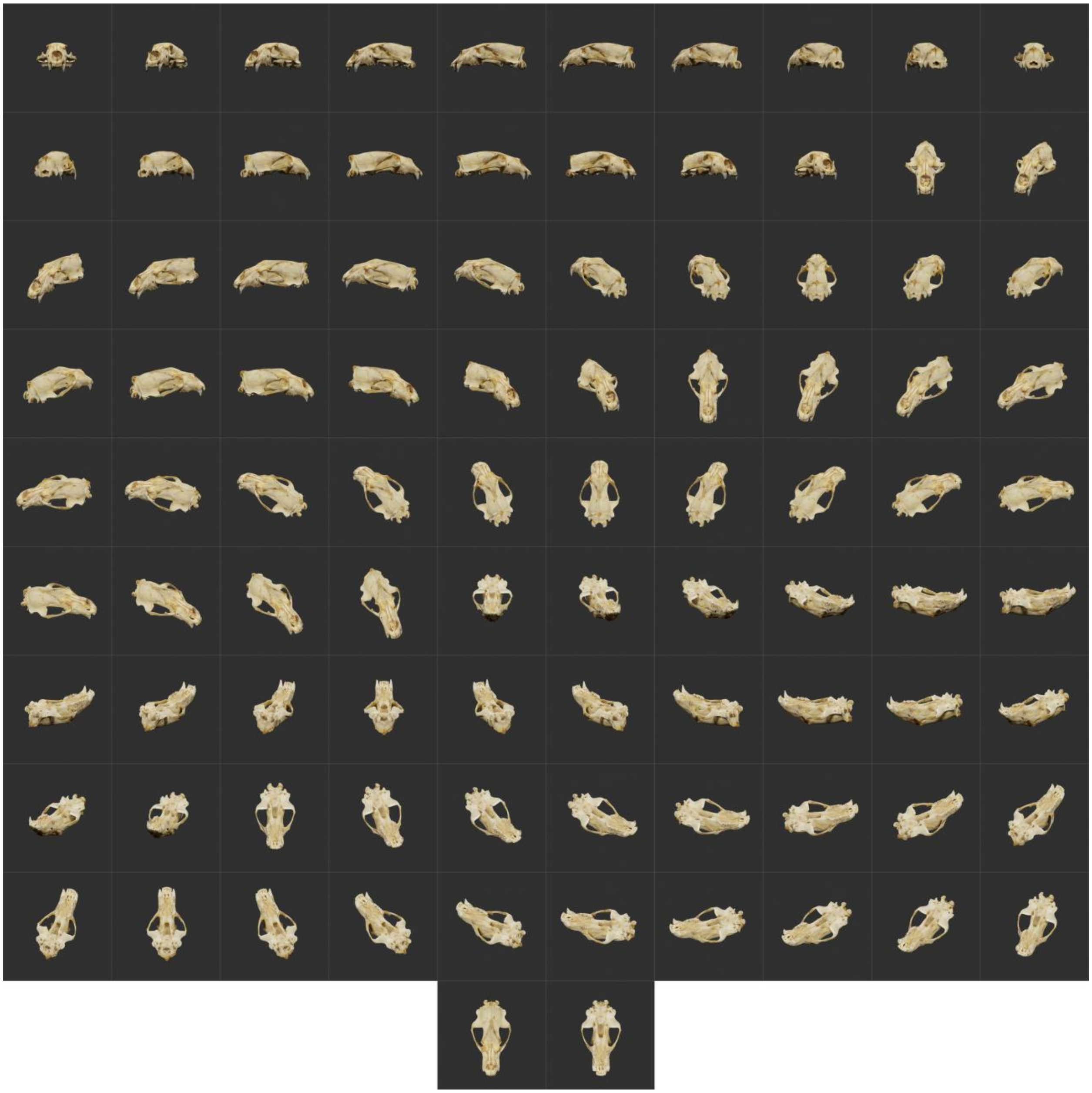

Fig S.3: A collage of synthetic images of a polar bear (*Ursus maritimus*) skull, showing all 92 imaging angles.

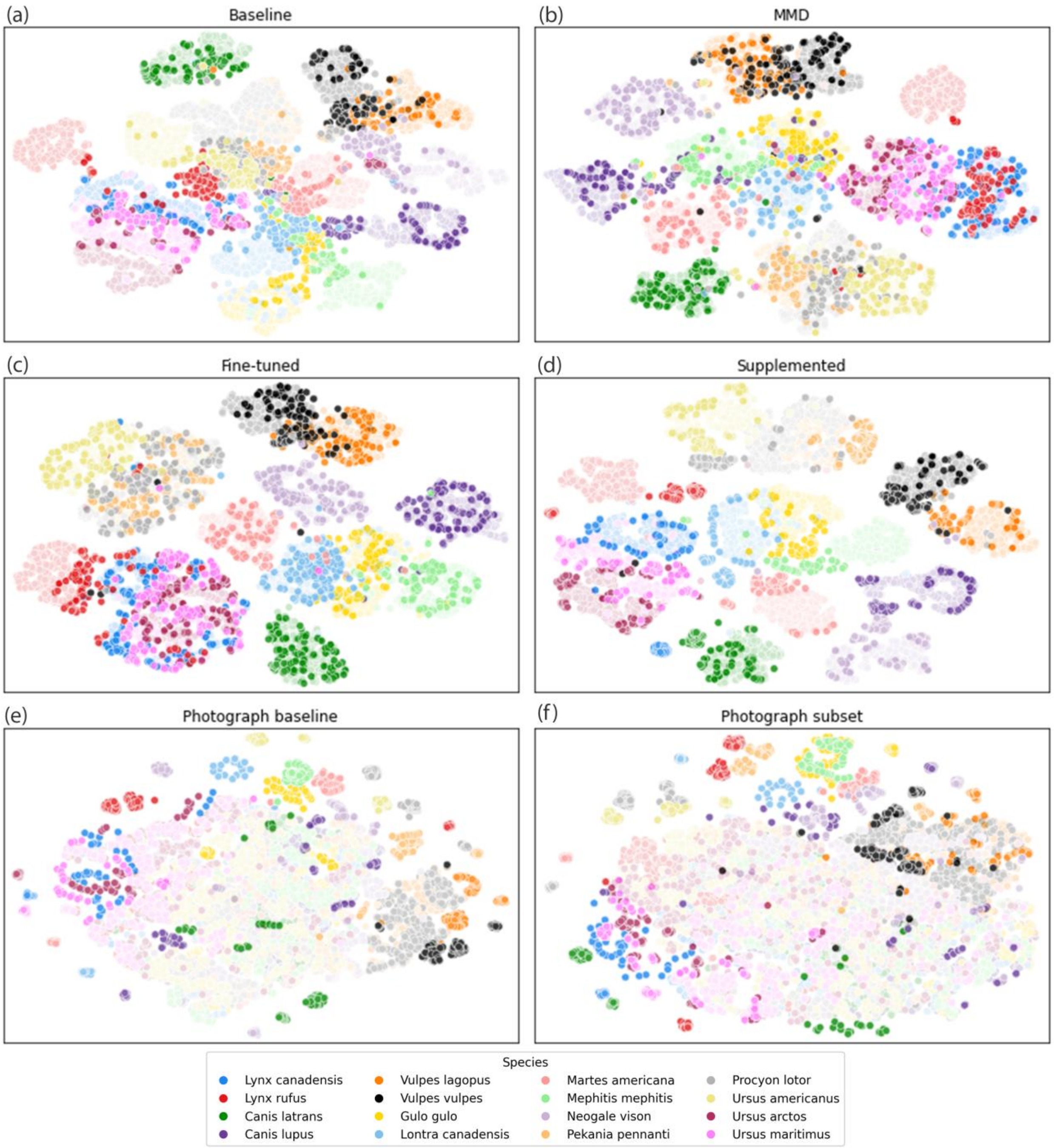

Fig S.4: Visualisation of the feature space of six skull classification models using t-SNE. Each t-SNE plot was generated from using the activations of the model's post-convolution flattened layer. Each species is represented by a unique colour. Translucent points represent synthetic images and opaque points represent photographs. All images were from the test dataset.

Tables

Table S.1: The carnivore species used for this study. All specimens are housed and curated by the Canadian Museum of Nature. The "Synthetic images" column shows example renders of 3D skull assets, as well as the number of images in the synthetic images training and testing datasets. The "Photographs" column shows examples of photographs of skull specimens, as well as the number of photos in the training, training subset, and testing photograph datasets. The photograph training subset images are part of the photograph training dataset, hence why the subset number is in square brackets.

**Family**	**Species (common name)**	**Synthetic images (training/testing)**	**Photographs (training[subset]/ testing)**
Felidae	*Lynx canadensis* (Canada lynx)	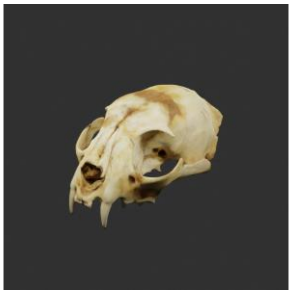 2208/552	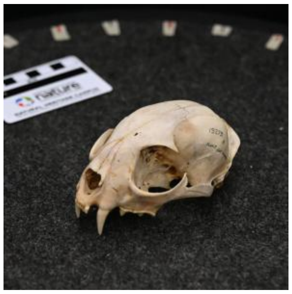 718[184]/184
Felidae	*Lynx rufus* (Bobcat)	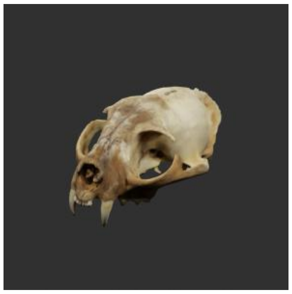 2208/552	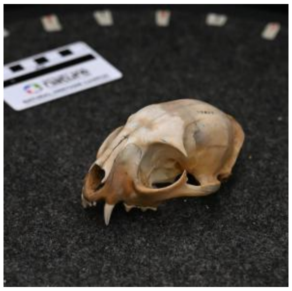 736[184]/184
Canidae	*Canis latrans* (Coyote)	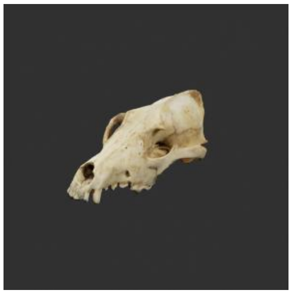 2208/552	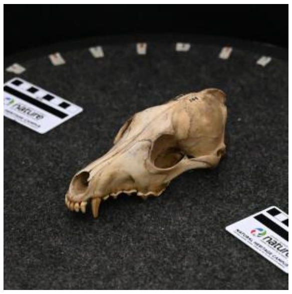 736[184]/184

	Canis lupus<br>(Grey wolf)	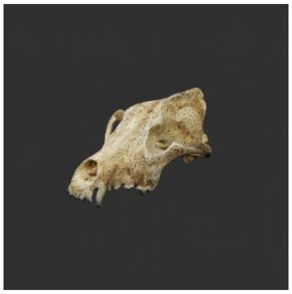<br>2208/552	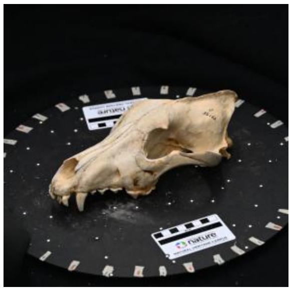<br>736[184]/184
	Vulpes lagopus<br>(Arctic fox)	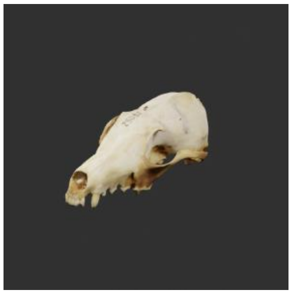<br>2208/552	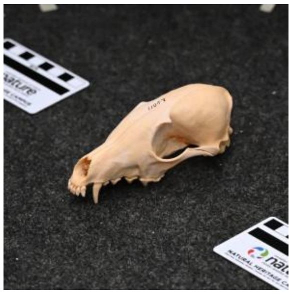<br>736[184]/184
Canidae	*Vulpes vulpes*<br>(Red fox)	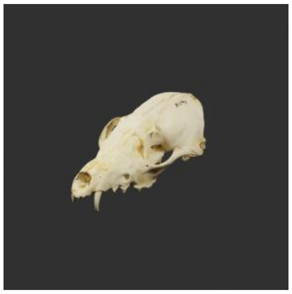<br>2208/460	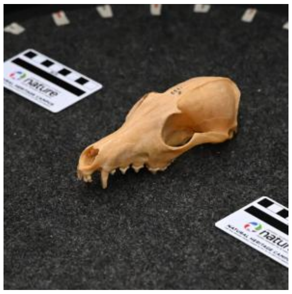<br>736[184]/184
Musteloidea<br>(Superfamily)	*Gulo gulo*<br>(Wolverine)	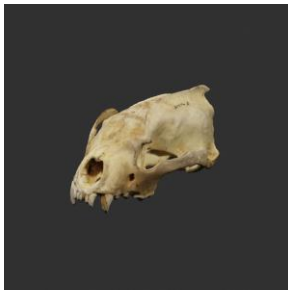<br>2208/552	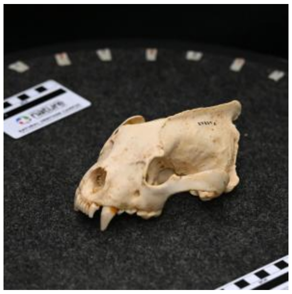<br>736[184]/184

<table>
<tr><td rowspan="2"></td><td>Lontra canadensis
(River otter)</td><td>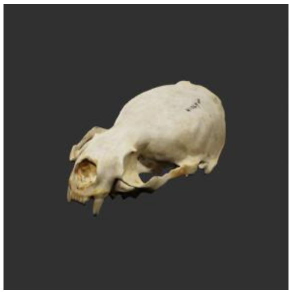
2208/552</td><td>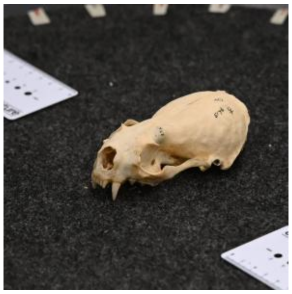
726[184]/184</td></tr>
<tr><td>Martes americana
(American marten)</td><td>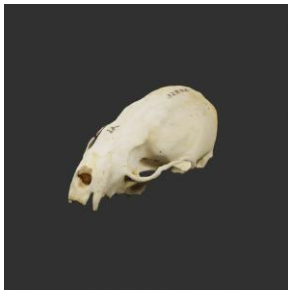
2208/552</td><td>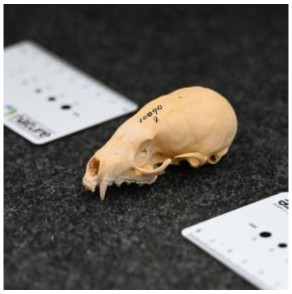
717[184]/184</td></tr>
<tr><td rowspan="2">Musteloidea
(Superfamily)</td><td>Mephitis mephitis
(Striped skunk)</td><td>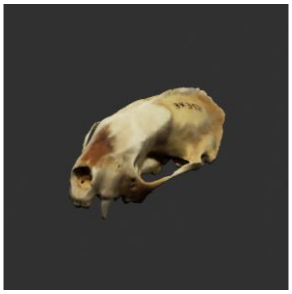
2208/552</td><td>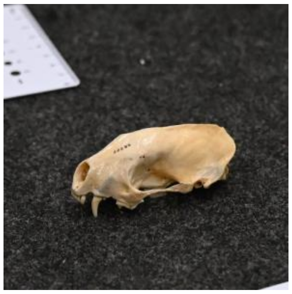
735[184]/184</td></tr>
<tr><td>Neogale vison
(American mink)</td><td>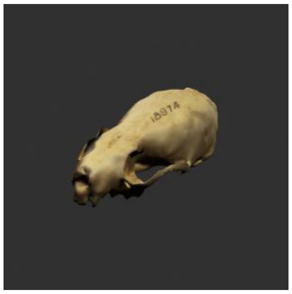
2208/552</td><td>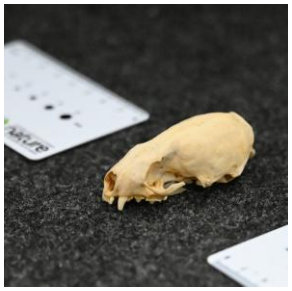
590[147]/148</td></tr>
</table>

	Pekania pennanti<br>(Fisher)	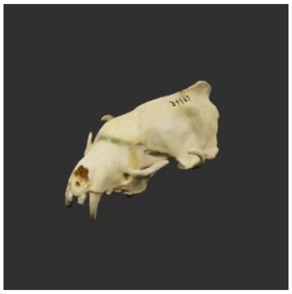<br>2208/552	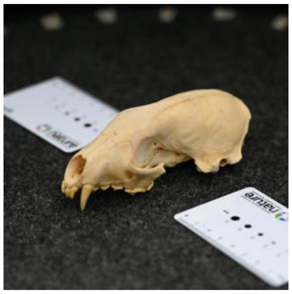<br>735[184]/184
	Procyon lotor<br>(Common raccoon)	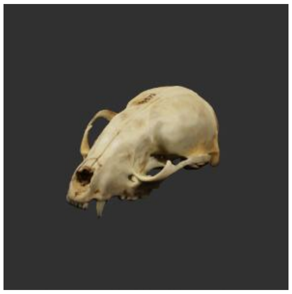<br>2208/552	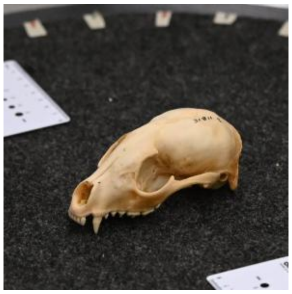<br>734[184]/184
Ursidae	*Ursus americanus*<br>(Black bear)	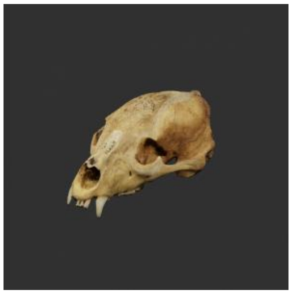<br>2208/552	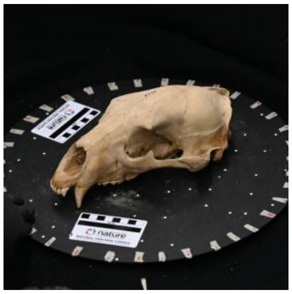<br>718[184]/184
	Ursus arctos<br>(Brown bear)	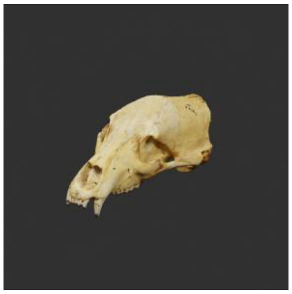<br>2208/552	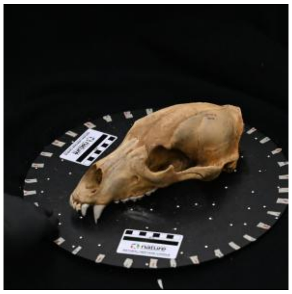<br>736[184]/182

	Ursus maritimus (Polar bear)	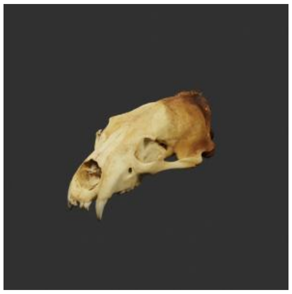 2208/552	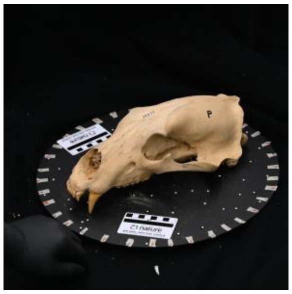 736[184]/184

Table S.2: Grad-CAM heatmap scoring scheme. The second column describes the subjective criteria used to assign a Grad-CAM each score. The third column, “Examples”, shows example Grad-CAMs for each score.

Score	Criteria	Examples
**3**	The heatmap appears red over the skull and blue over the background.	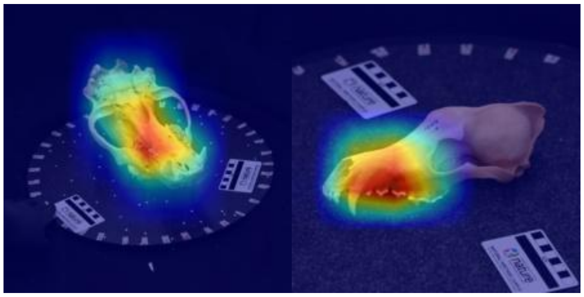
**2**	**Option 1**: The heatmap appears yellow/green over the skull and blue over the background.<br>**Option 2**: The heatmap appears red over the skull and yellow/green over a small background feature (e.g., a scale card)	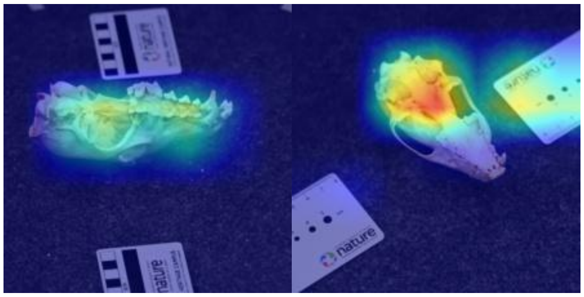
**1**	**Option 1**: The heatmap shows equal activation (i.e., red/yellow/green) over the skull and a small background feature (e.g., a scale card). The rest of the background is blue.<br>**Option 2**: The heatmap appears red over the skull and green over large areas of the image’s background.	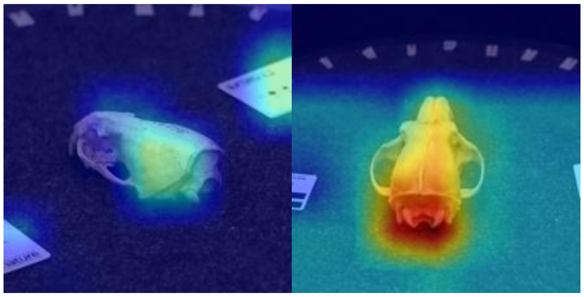
**0**	**Option 1**: The heatmap shows activation (i.e., red/yellow) over large areas of the image’s background.<br>**Option 2**: The heatmap shows higher activation over a background feature than over the skull.	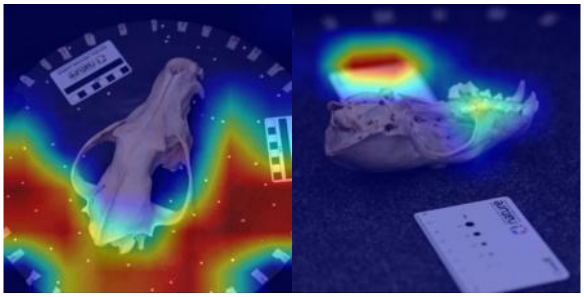
