# Supplementary material for: Leveraging synthetic data produced from museum specimens to train adaptable species classification models
Source: PLoS One. 2025 Sep 3;20(9):e0329482. doi: 10.1371/journal.pone.0329482 (PMC12407421; doi:10.1371/journal.pone.0329482)
Supplement: Attachment — Submitted filename: Plos Response.pdf [file pone.0329482.s002.pdf]

Response to reviewers

Reviewer

> Reviewer: *In line 201, you state that you scaled the images to 224x224 pixels for both training and testing. It seems to me that this low resolution could bias the performance of the model, since it could lead to loss of details in the images. Can you please explain why you decided to operate at this resolution, and why? Have you compared other resolutions, in order to decide the one with the best cost/effectiveness ratio? Please, provide some metrics.*

Response: We chose to rescale our images to this resolution because it is the resolution required by the VGG19 CNN architecture. The VGG19 architecture was built to receive 224x224 pixel images because this is the resolution of the images in the ImageNet dataset, a large image dataset used to benchmark CNN models. As a result, most state-of-the-art CNN architectures (ResNet, EfficientNet, DenseNet, etc.) are also designed to receive images scaled to 224x224, and this resolution has become somewhat of a standard in the field of computer vision. Some studies have tested the effect of image resolution on classification performance, and while an increase in resolution can improve performance, the benefits of increasing resolution begin to plateau at images larger than 128x128 pixels (Thambawita *et al.*, 2021; Haque *et al.*, 2023). Additionally, using higher-resolution images comes with significant computational costs.

We have now included a brief explanation for why we use this resolution in line 191.

Haque, M.I.U. *et al.* (2023) 'Effect of image resolution on automated classification of chest X-rays', *Journal of Medical Imaging*, 10(04). https://doi.org/10.1117/1.JMI.10.4.044503.

Thambawita, V. *et al.* (2021) 'Impact of Image Resolution on Deep Learning Performance in Endoscopy Image Classification: An Experimental Study Using a Large Dataset of Endoscopic Images', *Diagnostics*, 11(12), p. 2183. https://doi.org/10.3390/diagnostics11122183.

Editor

Editor: *Please ensure that your manuscript meets PLOS ONE's style requirements, including those for file naming.*

Response: We have made updates to the style/format of the manuscript to comply with PLOS ONE's style requirements.

Editor: *Please update your submission to use the PLOS LaTeX template.*

Response: We have converted our manuscript to LaTeX using PLOS's template. However, in doing this we discovered that the Table 1 from our original manuscript is not compatible with LaTeX. As a result, we moved it to the supplemental file (it is now S1 Table), and added a new figure (Fig 1) so example images of our 3D renders and photographs are still included in the main text of the manuscript.

> Editor: *In your manuscript, please provide additional information regarding the specimens used in your study. Ensure that you have reported human remain specimen numbers and complete repository information, including museum name and geographic location.*
>
> *If permits were required, please ensure that you have provided details for all permits that were obtained, including the full name of the issuing authority, and add the following statement:*
>
> *'All necessary permits were obtained for the described study, which complied with all relevant regulations.'*
>
> *If no permits were required, please include the following statement:*
>
> *'No permits were required for the described study, which complied with all relevant regulations.'*

Response: We have now included a data table in our repository containing collection catalog numbers for all specimens used in the study. No permits were required for our study. We have now included this statement in lines 114-115.

> Editor: *Please note that PLOS ONE has specific guidelines on code sharing for submissions in which author-generated code underpins the findings in the manuscript. In these cases, we expect all author-generated code to be made available without restrictions upon publication of the work.*

Response: We have made our code open source and accessible through a GitHub repository archived on Zenodo. We include a reference to this repository in the manuscript.

> Editor: *We note that you have provided funding information that is not currently declared in your Funding Statement. However, funding information should not appear in the Acknowledgments section or other areas of your manuscript. We will only publish funding information present in the Funding Statement section of the online submission form.*
> *Please remove any funding-related text from the manuscript and let us know how you would like to update your Funding Statement. Currently, your Funding Statement reads as follows:*

“JDB received a Mitacs Accelerate grant to fund this work. The grant does not have a specific grant number. Mitacs' website can be found at https://www.mitacs.ca/. The funders did not play any role in the study design, data collection, decision to publish, or preparation of the manuscript.”

Response: We have revised our acknowledgments section to remove the text referring to JDB’s funding. The acknowledgements section originally referred to a “Mitacs Elevate grant”, which is different from a “Mitacs Accelerate grant”, as noted in your comment. We would like to clarify that the text in the original acknowledgements section was incorrect, and JDB only received a Mitacs Accelerate grant. The original funding statement is therefore accurate, and I have noted this in our cover letter.

Editor: *Please include captions for your Supporting Information files at the end of your manuscript, and update any in-text citations to match accordingly.*

Response: We have implemented the requested changes.

Editor: *Please review your reference list to ensure that it is complete and correct.*

Response: We have reviewed our reference list to ensure that it is complete and correct.

Editor: *This MS deserves to be published on PLOS One. It is well written in a scientific form. The authors have to follow the suggestions of the reviewer to prepare your improved MS. In addition do not have to forget to add the authors of species names the first time that them are cited.*

Response: We appreciate the editor’s endorsement of our manuscript. We have responded to the reviewer’s comment, and included species authors when we first cite them in the manuscript.
